# Maternal BMI mediates the impact of crop-related agricultural work during pregnancy on infant length in rural Pakistan: a mediation analysis of cross-sectional data

**DOI:** 10.1186/s12884-019-2638-3

**Published:** 2019-12-17

**Authors:** Rebecca Pradeilles, Elizabeth Allen, Haris Gazdar, Hussain Bux Mallah, Azmat Budhani, Rashid Mehmood, Sidra Mazhar, Ayesha Mysorewala, Saba Aslam, Alan D. Dangour, Elaine Ferguson

**Affiliations:** 1 Department of Population Health, Faculty of Epidemiology and Population Health, London School of Hygiene & Tropical Medicine, London, UK; 2 School of Sport, Exercise and Health Sciences, Loughborough University, Loughborough, UK; 3 Department of Medical Statistics, Faculty of Epidemiology and Population Health, London School of Hygiene & Tropical Medicine, London, UK; 4 Collective for Social Science Research, Karachi, Pakistan

**Keywords:** Agricultural work, Nutritional status, Women, Infant, Rural Pakistan, Mediation analysis

## Abstract

**Background:**

Stunted growth in early infancy is a public health problem in low-and-middle income countries. Evidence suggests heavy agricultural work during pregnancy is inversely associated with maternal body mass index (BMI) and infant birth weight in low- and middle-income countries; but pathways linking agricultural work to length-for-age Z-scores (LAZ) in early infancy have not been examined. This study aimed to investigate the relationship between agricultural work during pregnancy, post-natal maternal BMI and LAZ among young infants in rural Pakistan; and explored whether maternal BMI mediated the relationship between agricultural work and infant LAZ.

**Methods:**

A cross-sectional survey was conducted from December 2015 to January 2016 in rural Sindh, Pakistan. Mother-infant dyads were recruited via systematic random cluster sampling at 2–12 weeks’ post-partum (*n* = 1161). Anthropometric measurements (maternal and infant height/length and weight) and questionnaire data were collected. Multivariable linear regression and structural-equation based mediation analyses were used to examine associations of agricultural work during pregnancy with maternal BMI and infant LAZ.

**Results:**

During pregnancy, women reported engaging in livestock-related work (57.0%), crop-related work (42.7%), and cotton harvesting (28.4%). All three forms of agricultural work were negatively associated with maternal BMI (β = − 0.67 [− 1.06; − 0.28], β = − 0.97 [− 1.51; − 0.48]; and β = − 0.87 [− 1.33; − 0.45], respectively). Maternal engagement in cotton harvesting alone was negatively associated with infant LAZ after controlling for confounding factors. The total negative effect of cotton harvesting on infant LAZ was − 0.35 [− 0.53; − 0.16]. The indirect effect of maternal BMI on infant LAZ was − 0.06 [− 0.08; − 0.03], revealing that 16% (− 0.06/− 0.35) of the relationship between cotton harvesting and infant LAZ, after adjustment, was mediated via maternal BMI.

**Conclusion:**

These results underscore a need to reduce labour-intensive agricultural workload demands during pregnancy, especially in cotton harvesting, to reduce risks of negative maternal energy balance and poor growth outcomes in early infancy.

## Background

Despite economic growth in recent years, levels of childhood under-nutrition in Pakistan remain among the highest globally [1]. Maternal nutrition prior to and during pregnancy, environmental exposures and lifestyle behaviours increase the risk of intrauterine growth restriction and preterm birth, leading to childhood undernutrition [2, 3]. Among lifestyle behaviours, employment in agriculture is particularly relevant in Pakistan because agriculture is the most common occupation among employed women [4]; and their roles often involve long hours of labour-intensive agricultural work [5].

While paid agricultural employment can benefit rural women by increasing their social status, decision-making ability and purchasing power, excessive energy expenditure during periods of intensive agriculture work and exposure to toxic chemicals, such as pesticides, can compromise maternal nutrition and health [3, 6]. Pregnant women involved in labour-intensive work such as cotton harvesting are at particularly high risk. The physically demanding work, long hours and potential exposure to chemical hazards can have negative effects on the health of pregnant women and the development of their foetuses [2, 7, 8].

Evidence for an association between physical activity during pregnancy and birth outcomes is inconsistent [2, 9, 10]. Women involved in physically demanding work, especially in late pregnancy, have a small increased relative risk of a preterm birth [10]; whereas results are inconsistent for small-for-gestational-age births [2, 9, 10]. However, most studies were conducted on populations living in North America and Europe, where the intensity of physical activity is likely lower than rural women in low- and middle-income countries (LMICs). The few studies that have examined the impact of labour-intensive agricultural work on maternal nutrition and pregnancy outcomes in LMICs show labour-intensive agriculture reduces birth weight and/or weight gain in pregnancy; although its impact may depend on the timing of the exposure (i.e., third trimester) and nutritional status of the pregnant woman [8, 11, 12].

Previous studies have examined direct pathways between maternal agricultural workload and pregnancy outcomes only, without considering mediating factors and the different types of agriculture work in which rural women are involved. The current study examines the direct and indirect relationships between livestock or crop-related agricultural activities performed during pregnancy, maternal body mass index (BMI) and infant length-for-age Z-score (LAZ) in a sample of mother-infant dyads living in rural Sindh, Pakistan. We use structural equation modelling to provide an interpretative modelling structure that accounts for interdependencies between variables in the models. An understanding of inter-linking pathways is essential for the design of effective policies or intervention practices.

## Methods

### Survey design and setting

The Women’s Work in Agriculture and Nutrition (WWN) study is a longitudinal study of mother-infant dyads living in irrigated rural areas of Sindh province, Pakistan. Here we use data from the baseline cross-sectional survey, conducted from December 2015 to February 2016.

### Sampling

A sample size of 1000 dyads was calculated to detect a difference in maternal BMI of 0.18 for every additional hour worked with 80% power at a 5% level of significance [4]. This sample size provides adequate power to explore factors associated with maternal and infant nutritional status and to perform statistical mediation analysis [13]. Further information on the sampling strategy is provided in Additional file 1.

Participants were selected via systematic random cluster sampling. Initially, administrative villages with perennial canal irrigation were selected; villages with a population below the 10th and above the 90th percentiles of estimated village sizes were excluded, and random sampling was used to select villages to provide the estimated sample size of 1000 mother-infant dyads. Villages with perennial canal irrigation were chosen as the study site because women in these villages are frequently involved in commercial agriculture, including cotton harvesting. All dyads in selected villages were invited to participate in the study if: (i) the infant was a singleton birth ≥2 weeks and ≤ 12 weeks of age on the day of the first interview; healthy without congenital deformations that would impact on their ability to eat and (ii) the primary caregiver (i.e. the biological mother) intended to reside in the study area over the next 10 months.

### Questionnaire and spot observations

An interviewer-administered questionnaire was collected from mothers using electronic data capture (Samsung tab-4) to obtain information relating to: socio-demographic characteristics, household food insecurity, health, maternal 24-h food consumption and maternal agriculture work history during pregnancy (Additional file 2). The questionnaire took approximately 60 min to complete. Spot observations were also performed to record housing materials and the hygienic conditions of the environment.

### Anthropometric assessment

Two serial measurements of maternal and infant weight and height/length were collected, following standard procedures [14], by trained fieldworkers who were selected based on their technical error of measurement (TEM) results following a 5-day training programme. After removing shoes and heavy clothing, maternal weight was measured to the nearest 0.5 kg using digital electronic scales (Tanita digital bathroom scale); maternal height was measured to the nearest 0.1 cm using a portable stadiometer (Seca 213); infant weight was measured to the nearest 0.01 kg using digital electronic scales (LAICA weight scale for babies) and infant length was measured to the nearest 0.1 cm using an infantometer (Seca 416). A third measurement was taken if the difference between the first two measurements was above a pre-defined threshold (i.e. > 0.7 cm for maternal height and infant length; > 0.5 kg for maternal weight; > 0.1 kg for infant weight); and an average of the two closest measurements was calculated. For women who refused to remove heavy jewellery and/or clothing (*n* = 134; 11.7%), their measured weight was reduced by 0.5 kg for heavy jewellery and 0.5 kg for heavy clothing. No adjustments for clothing were made to infant weight.

### Data management and analyses

#### Anthropometric characteristics

Maternal BMI post-pregnancy was calculated (weight (kg)/(height (m^2^)); and women were classified as underweight, normal weight, overweight and obese using age-specific international cut-off points for adolescents aged less than 18 years [15, 16]; and the World Health Organization (WHO) adult cut-offs for women aged 18 years or above [17].

Infant LAZ and weight-for-length (WLZ) were generated using the WHO growth standards [14]. Data for infants with biologically implausible anthropometric results were excluded (i.e., those with z-scores <− 6 SD or > + 6 SD for LAZ and those with z-scores <− 5 SD or > + 5 SD for WLZ) [14]. Infants were classified as stunted or wasted if their z-scores for LAZ or WLZ were < − 2 SD, respectively [14].

#### Socio-demographic characteristics

A household wealth index was created using factor analysis applied to proxy indicators of the household environment (ownership of consumer durables; house ownership; land ownership; main source of energy for cooking; livestock ownership; electricity; source of drinking water and type of toilet facilities; number of rooms used for sleeping; type of materials used for floors, the roof and walls). Socio-economic status (SES) quintiles were created; and internal validity was checked by assessing ownership of durable assets and housing characteristics by SES quintile. Maternal education was not included in the creation of the household wealth index because of its known independent effect on nutrition and health outcomes.

#### Food insecurity and maternal dietary diversity

To measure food insecurity, answers to questions on anxiety and uncertainty about the household food supply and insufficient food quality experienced in the past 30 days were collected [18]. Binary variables (yes/no) were created to capture the proportion of households who ate a limited variety of foods due to a lack of resources in the past 30 days and to capture the proportion of households who worried about not having enough food in the past 30 days. The Household Food Insecurity Access Scale was not generated because we collected only a sub-set of the questions to reduce the length of the questionnaire and respondent burden.

To measure maternal dietary diversity, a single semi-qualitative 24 h dietary recall was used. The Minimum Dietary Diversity score (ordinal variable) was then generated for women of reproductive age, using reported intakes of foods and beverages during the past 24-h [19].

#### Agricultural work data

Binary variables (yes/no) were created for reported engagement during pregnancy in livestock-related agricultural activities, any crop-related agricultural activities (including cotton harvesting), and cotton harvesting alone. The livestock-related activities included fodder collection/preparation/chopping, animal washing, milking animals, providing care to animals, grazing animals, giving water to animals and egg collection. The crop-related activities included sowing, transplanting, digging, weeding, applying fertilizer, grain harvesting, vegetable harvesting and cotton harvesting. Cotton harvesting, which is almost exclusively done by women in this part of Pakistan, was examined separately because it involves particularly long hours of intensive work under the sun during the summer/autumn months (July to November).

### Statistical analyses

Hypothesized models of pathways related to maternal BMI and infant size were drawn based on the available literature and discussion with experts (Additional file 3). These diagrams were drawn using the DAGitty software and constitute directed acyclic graphs (DAG). These DAGs provide a means of visually identifying common causes and effects in a set of variables within a multivariable model and are used to identify variables to be included in multivariable analysis to estimate the total effect of pre-specified exposures of interest [20].

Associations of agricultural-related work with maternal BMI and infant LAZ were examined using univariable and multivariable linear regressions. We restricted the analysis to LAZ (in contrast to WLZ) as LAZ was more likely to reflect maternal conditions (i.e. agricultural work) during pregnancy. WLZ is more susceptible to any adverse exposures in the immediate environment after birth and may mask the effects of maternal agricultural work in pregnancy.

The variables included in the multivariable models were retained based on the information provided by the DAGs (Additional file 3). Some pre-specified covariates were included in the model due to their known effect on maternal BMI (i.e. maternal age and number of days post-partum) and infant growth (i.e. infant age and sex, maternal height) [21]. We adjusted for the number of weeks post-partum (2–12 weeks) at which maternal weight was collected to address potential bias in associations with maternal BMI. The multivariable analyses were conducted on the complete case sample.

Structural-equation models were used to test whether maternal BMI (post pregnancy) mediated the association between agricultural work during pregnancy and infant LAZ at 2–12 weeks of age. The total effect was decomposed into direct and indirect effects. We ensured that the temporality of the variables in the model was respected. Maternal workload during pregnancy precedes both maternal BMI post-pregnancy (mediating factor) and infant LAZ at 2–12 weeks (outcome). Both maternal BMI and infant LAZ were measured around the same time and so it is difficult to prove that maternal BMI precedes infant LAZ but maternal BMI post-pregnancy was used as a proxy for maternal nutritional status during pregnancy. From a biological point of view, maternal BMI would predict infant LAZ rather than vice versa. SEM models were driven by both our initial hypothesis and by the significance of the initial associations.

Non-parametric bootstrapping was used to estimate confidence intervals (CIs) and corresponding *p*-values in the univariable and multivariable analyses [22]. Robust standard errors were used to account for clustering at the village level. All analyses were conducted using Stata/IC (version 14.1). The type I error risk was set at 0.05.

## Results

The sampling strategy identified 1729 potentially eligible mother-infant dyads (from 62 sampled villages), of which 568 (32.8%) did not meet the inclusion criteria or refused to participate (less than 1% refused), resulting in a final sample of 1161 dyads (Additional file 4). Interviewer-administered questionnaires were completed by 99.5% of respondents (*n* = 1155). Anthropometric measurements were provided by 98.7% (*n* = 1146) and 98.4% (*n* = 1143) of women and infants, respectively, and LAZ data from nine infants were excluded as outliers (Additional file 4).

The TEM values of the three teams of anthropometrists for maternal and infant height/length and weight, respectively, ranged from 0.3 to 3.5%.

Overall 99.7% of the women in the study were married and 81.0% of them had no formal education (vs. 48.0% for men) **(**Table 1**)**. The median age for women was 27.0 years. Household size ranged from 3 to 36 members; and the median parity of respondents was three. Overall 59.3% of the sample reporting being worried about not having enough food in the past 30 days, and one third of households (32.5%) received government income support. More than half (52.5%) of the female respondents were engaged in the agricultural sector. Livestock-related and crop-related activities, during pregnancy, were performed by 57.0 and 42.7% of women, respectively. The most common crop-related activities performed during pregnancy (as a percentage of all women) were: cotton harvesting (28.4%), weeding (16.8%), and grain harvesting (14.4%). The most common livestock-related activities performed during pregnancy (as a percentage of all women) were: giving water to animals (41.9%), fodder preparation (27.3%) and milking animals (22.9%).

**Table 1:** Socio-demographic characteristics, agriculture-related work practices and food security

	n	Median (IQR) / %
Infant age (m)	1134	1.6 (1.0, 2.1)
Infant sex (%)	1134	
Male	569	50.2
Maternal age (y)	1096	27.0 (23.0, 32.0)
Marital status (%,)	1138	
Married	1134	99.7
Maternal education (%)	1140	
No formal education	923	81.0
Primary school education	13	11.8
> Primary school education	82	7.2
Paternal education (%)	1130	
No formal education	543	48.0
Primary school education	212	18.8
> Primary school education	375	33.2
Maternal occupation (%)	1126	
Unemployed	377	33.5
Agriculture related employment	591	52.5
Non-agriculture-related employment	158	14.0
Paternal occupation (%)	1142	
Unemployed	34	3.0
Agriculture related employment	585	51.2
Non-agriculture-related employment	523	45.8
Maternal agriculture work during pregnancy (%)	1134	
Livestock-related work	647	57.0
Crop-related work `	484	42.7
Cotton harvesting	322	28.4
Food security (%)	1139	
Worried there was insufficient food in last 30 d	675	59.3
Ate a limited variety of food in last 30 d	713	62.7
Cash transfer from the Benazir Income Support	1137	
Program (%, yes)	369	32.5

At a median age of 6.9 weeks, a high percentage of the infants were stunted (45.0%) and/or wasted (12.4%). Close to one quarter of mothers were underweight (21.6%); although 13.1% were overweight or obese **(**Table 2**)**.

**Table 2:** Anthropometric Characteristics

	n	Median (IQR) / %
Infant		
Length (cm)	1134	52.5 (50.0, 55.1)
Weight (kg)	1134	3.75 (3.2, 4.4)
Length-for-age Z-score	1134	−1.82 (−2.7, −0.9)
% stunted (LAZ < -2 Z-score)	515	45.4
Weight-for-length Z-score	1098	−0.49 (− 1.3; 0.2)
% wasted (WLZ < -2 Z-score)	136	12.4
Mother		
Height (cm)	1146	152.6 (148.9, 156.2)
Weight (kg)	1146	47.8 (43.0, 53.5)
Body mass index (kg/m^2^)	1146	20.5 (18.7, 22.6)
% Underweight (BMI < 18.5)	248	21.6
% Normal weight (18.5 < BMI < 25)	48	65.3
% Overweight/obese (BMI ≥ 25)	150	13.1

### Maternal BMI

Crop-related agriculture work, cotton harvesting or livestock-related work conducted during pregnancy were all negatively associated with maternal BMI even after adjusting for confounding factors (β **= −** 1.00 kg/m^2^ [− 1.53; − 0.47]; β = − 0.88 kg/m^2^ [− 1.35; − 0.42] and β = − 0.60 kg/m^2^ [− 1.03; − 0.17], respectively) **(**Table 3**)**. Other factors positively associated with maternal BMI in the multivariable analysis were maternal age, maternal education and the household wealth quintiles (Table 3).

**Table 3:** Factors associated with maternal BMI from the univariable and multivariable regression analysis

		Model 1 (*n* = 1146)	Model 2 (*n* = 1064)	Model 3 (*n* = 1064)	Model 4 (*n* = 1064)
	n	β (95% CI) ^1^	β (95% CI) ^2^	β (95% CI) ^2^	β (95% CI) ^2^
Agricultural related factors					
Any crop-related work during pregnancy	1134	*p < 0.0001*	*p < 0.0001*		
No	650	Ref^3^	Ref		
Yes	484	−1.59 (−2.10; −1.08)	−1.00 (−1.53; −0.47)		
Cotton harvesting during pregnancy	1134	*p < 0.0001*		*p < 0.0001*	
No	812	Ref		Ref	
Yes	322	−1.49 (−2.03; −0.95)		−0.88 (− 1.35; − 0.42)	
Livestock-related work during pregnancy	1134	*p < 0.0001*			*p = 0.006*
No	487	Ref			Ref
Yes	647	−0.88 (−1.32; − 0.44)			−0.60 (−1.03; − 0.17)
Individual level factors					
		*p < 0.0001*	*p < 0.0001*	*p < 0.0001*	*p < 0.0001*
Maternal age	1096	0.10 (0.06; 0.14)	0.11 (0.07; 0.15)	0.05 (0.07; 0.15)	0.11 (0.07; 0.15)
		*p < 0.0001*			
Maternal parity	1140	0.29 (0.19; 0.39)			
		*p < 0.0001*			
Maternal dietary diversity	1138	0.62 (0.43; 0.81)			
		*p = 0.22*	*p = 0.41*	*p = 0.49*	*p = 0.49*
Number of days post-partum	1140	−0.01 (− 0.02; 0.00)	−0.00 (− 0.01; 0.01)	−0.00 (− 0.01; 0.01)	−0.00 (− 0.01; 0.01)
Household level factors					
Maternal education	1140	*p < 0.0001*	*p = 0.0004*	*p = 0.0004*	*p = 0.0002*
Not educated	923	Ref	Ref	Ref	Ref
Primary school	135	1.18 (0.29; 2.07)	0.71 (−0.06; 1.48)	0.70 (− 0.09; 1.49)	0.69 (− 0.09; 1.47)
Middle, secondary and higher education	82	2.53 (1.53; 3.53)	1.42 (0.59; 2.26)	1.52 (0.65; 2.39)	1.49 (0.66; 2.32)
Household wealth quintiles	1108	*p < 0.0001*	*p < 0.0001*	*p < 0.0001*	*p < 0.0001*
Poorest	221	Ref	Ref	Ref	Ref
Poor	223	0.52 (−0.17; 1.23)	0.41 (− 0.26; 1.09)	0.46 (− 0.21; 1.13)	0.54 (− 0.18; 1.27)
Middle	222	1.77 (0.97; 2.56)	1.37 (0.58; 2.16)	1.47 (0.70; 2.24)	1.63 (0.79; 2.47)
Wealthy	221	1.74 (1.03; 2.451)	1.18 (0.53; 1.83)	1.24 (0.59; 1.90)	1.44 (0.73; 2.15)
Wealthiest	221	2.96 (2.23; 3.69)	2.33 (1.68; 2.98)	2.40 (1.75; 3.05)	2.53 (1.86; 3.19)
Maternal occupation	1126	*p = 0.001*			
Not working	377	Ref			
Non-agricultural related work	158	0.14 (−0.71; 1.01)			
Agricultural related work	591	−1.15 (−1.85; − 0.46)			
Worry about not having enough food in the past 30 days	1139	*p = 0.001*			
No	464	0.89 (0.39; 1.40)			
Yes	675	Ref			
Eating a limited variety of food in the past 30 days	1137	*p < 0.0001*			
No	424	1.00 (0.49; 1.52)			
Yes	713	Ref			
Cash-aid from BISP	1137	*p = 0.74*			
No	768	Ref			
Yes	369	0.08 (−0.39; 0.55)			

^1^Unadjusted coefficients from univariable linear regression, ^2^Adjusted coefficients from multivariable linear regression (adjusted for maternal age, number of days post-partum, maternal education, and household socio-economic status), ^3^Reference

### Infant LAZ

Both crop-related agricultural work and cotton harvesting during pregnancy, were negatively associated with infant LAZ in the univariable analysis **(**Table 4**)**; however, only the relationship between cotton harvesting and infant LAZ remained significant after adjusting for potential confounding factors (i.e. household wealth index, maternal education, paternal education, maternal height, infant age and sex) (β = − 0.35z [− 0.55: − 0.14]). Livestock-related work during pregnancy was not associated with infant LAZ (β = − 0.08z [− 0.28; 0.11]). Other factors significantly associated with LAZ were infant sex and maternal height.

**Table 4:** Factors associated with infant growth (defined as length-for-age z-scores) from the univariable and multivariable regression analysis

		Model 1 (*n* = 1134)	Model 2 (*n* = 1083)	Model 3 (*n* = 1083)	Model 4 (*n* = 1083)
	n	β (95% CI) ^1^	β (95% CI) ^2^	β (95% CI) ^2^	β (95% CI) ^2^
Agriculture related factors					
Any crop-related work during pregnancy	1122	*p = 0.005*	*p = 0.06*		
No	644	Ref^3^	Ref		
Yes	478	−0.26 (−0.44; − 0.08)	− 0.19 (− 0.38; 0.01)		
Cotton harvesting during pregnancy	1122	*p < 0.0001*		*p = 0.001*	
No	803	Ref		Ref	
Yes	319	−0.39 (− 0.58; − 0.19)		− 0.35 (− 0.55; − 0.14)	
Livestock-related work during pregnancy	1122	*p = 0.39*			*p = 0.39*
No	483	Ref			Ref
Yes	639	−0.08 (− 0.26; 0.10)			−0.08 (− 0.28; 0.11)
Individual level factors					
		*p = 0.67*	*p = 0.49*	*p = 0.57*	*p = 0.57*
Infant age	1134	0.03 (−0.12; 0.19)	0.06 (−0.10; 0.22)	0.04 (− 0.11; 0.20)	0.04 (− 0.11; 0.20)
Sex of the infant	1134	*p = 0.11*	*p = 0.008*	*p = 0.009*	*p = 0.009*
Male	569	Ref	Ref	Ref	Ref
Female	565	0.13 (−0.03; 0.28)	0.20 (0.05; 0.35)	0.19 (0.05; 0.34)	0.20 (0.05; 0.35)
		*p < 0.0001*			
Maternal parity	1128	0.09 (0.05; 0.13)			
		*p < 0.0001*			
Maternal BMI	1134	0.07 (0.05; 0.09)			
		*p < 0.0001*	*p < 0.0001*	*p < 0.0001*	*p < 0.0001*
Maternal height	1134	0.06 (0.05; 0.08)	0.06 (0.05; 0.08)	0.06 (0.05; 0.08)	0.06 (0.05; 0.08)
Household level factors					
Maternal education	1128	*p = 0.004*	*p = 0.23*	*p = 0.22*	*p = 0.21*
Not educated	912	Ref	Ref	Ref	Ref
Primary school	134	0.12 (−0.15; 0.38)	−0.01 (−0.29; 0.27)	−0.00 (−0.28; 0.27)	0.00 (− 0.27; 0.28)
Middle, secondary and higher education	82	0.45 (0.18; 0.72)	0.25 (−0.08; 0.59)	0.26 (−0.07; 0.59)	0.28 (− 0.06; 0.62)
Paternal education	1118	*p = 0.08*	*p = 0.26*	*p = 0.29*	*p = 0.21*
Not educated	536	Ref	Ref	Ref	Ref
Primary school	210	0.03 (−0.20; 0.26)	0.03 (− 0.19; 0.25)	0.02 (− 0.20; 0.23)	0.04 (− 0.18; 0.26)
Middle, secondary and higher education	372	0.23 (0.02; 0.45)	0.17 (−0.03; 0.37)	0.16 (− 0.04; 0.35)	0.18 (− 0.02; 0.38)
Household SES quintiles	1096	*p = 0.04*	*p = 0.31*	*p = 0.43*	*p = 0.22*
Poorest	219	Ref	Ref	Ref	Ref
Poor	221	−0.01 (−0.32; 0.30)	−0.02 (− 0.33; 0.29)	−0.02 (− 0.32; 0.28)	0.01 (− 0.29; 0.31)
Middle	220	0.20 (− 0.09; 0.49)	0.09 (− 0.19; 0.37)	0.07 (− 0.21; 0.35)	0.13 (− 0.15; 0.41)
Wealthy	216	0.35 (0.05; 0.65)	0.22 (− 0.09; 0.53)	0.19 (− 0.12; 0.51)	0.28 (− 0.02; 0.58)
Wealthiest	220	0.27 (− 0.02; 0.56)	0.00 (− 0.32; 0.32)	−0.01 (− 0.34; 0.31)	0.06 (− 0.25; 0.36)
Maternal occupation	1114	*p = 0.02*			
Not working	374	Ref			
Non-agriculture related work	157	−0.24 (−0.42; − 0.06)			
Agriculture related work	583	−0.24 (− 0.53; 0.05)			
Cash-aid from BISP	1125	*p = 0.24*			
No	762	Ref			
Yes	363	0.10 (−0.07; 0.26)			

^1^Unadjusted coefficients from univariable linear regression, ^2^Adjusted coefficients from multivariable linear regression (adjusted for infant age, sex of the infant, maternal height, maternal education, paternal education, household socio-economic status), ^3^Reference

### Structural equation models

**Fig. 1:**
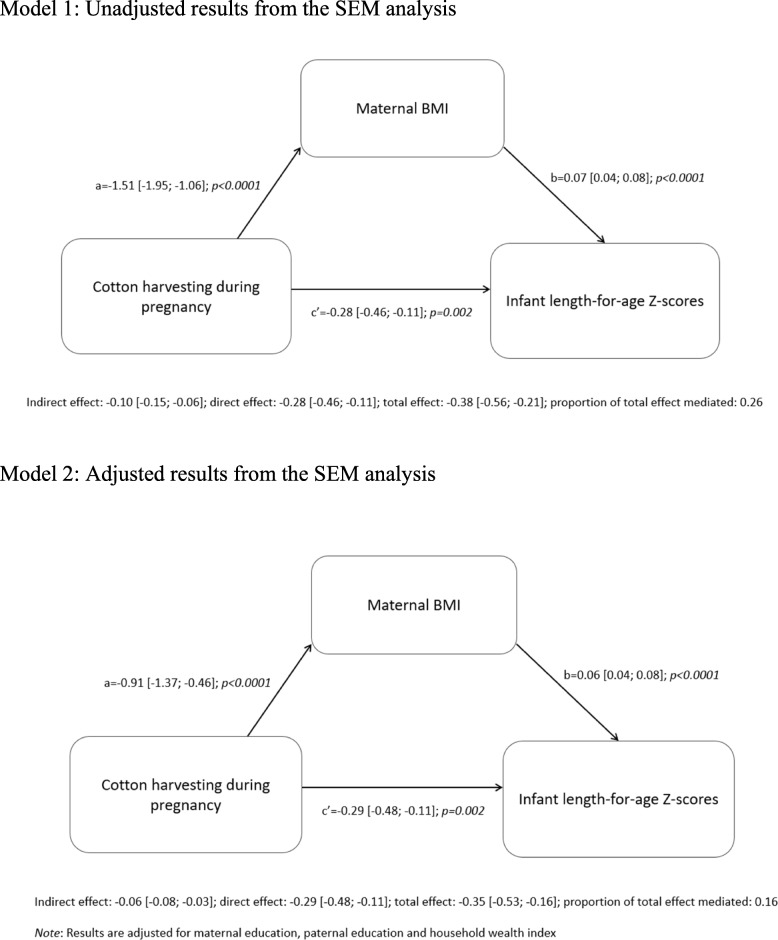
Structural-equation models assessing the relationship between cotton harvesting and infant LAZ via maternal BMI

Only cotton harvesting was associated with both maternal BMI and infant LAZ. Therefore, structural equation models were used to assess whether maternal BMI mediated the relationship between cotton harvesting and infant LAZ. This showed that cotton harvesting during pregnancy, was associated with direct, indirect and total (i.e. direct + indirect) effects on infant LAZ of − 0.28z [− 0.46; − 0.11], − 0.10z [− 0.15; − 0.06], and-0.38z [− 0.56; − 0.21] respectively, suggesting that maternal BMI mediated 26% of the relationship between cotton harvesting and infant LAZ **(**Fig. 1**)**. This was reduced to 16% after adjusting for potential confounders (household wealth index, maternal education and paternal education) (Fig. 1).

## Discussion

Results from this study show a very high prevalence of stunting among 2–12 week old infants, in rural irrigated areas of Sindh, compared with the national average for infants < 6 months of age (45 vs 26%) and other LMICs [1, 4]. It suggests the intrauterine environment contributes to poor growth outcomes in this region of Pakistan [23]. Our results also show that labour-intensive crop-related agriculture workloads during pregnancy, particularly cotton harvesting, contribute to these low infant LAZ and to low maternal BMI. We have also shown that maternal BMI mediates the association between cotton harvesting, during pregnancy, and infant LAZ (an indirect contribution of 16%).

These results are consistent with previous studies in India, which show that agricultural work during pregnancy is negatively associated with birth size [12, 24] and that adverse effects were most pronounced in under-nourished women who had low pregnancy weight gains [12].

However, they go beyond results from these previous studies by showing the indirect contribution of maternal nutritional status on infant LAZ, as distinct from other factors related to labour-intensive agricultural work (i.e., cotton harvesting). They suggest that even though reducing maternal under-nutrition is important for improving infant LAZ, addressing other factors related to cotton harvesting, such as long hours of labour-intensive agriculture work or exposure to chemical residue, is critical for reducing the high prevalence of early infant stunting in rural Sindh.

These results are not surprising. Intensive exercise in mid-to-late pregnancy, especially when it involves bending and lifting, can reduce placental function and blood flow, and thereby affect prenatal growth and early infant size [25]. Peak foetal length velocity occurs during the second trimester and organ maturation and weight gain occur during the third trimester. Environmental exposure to pesticides during cotton harvesting is also known to compromise the health of women in Pakistan [6], which in turn might compromise foetal development.

Previous studies suggest the timing of exposure to intensive agriculture work during pregnancy is important. They have shown that birth outcomes are negatively affected when intensive agriculture work occurs in the last trimester of pregnancy [8, 24]. In our study, 40% of women were involved in cotton harvesting only in their first and second trimester of pregnancy. This suggests that we may have under-estimated the overall impact of cotton harvesting on infant LAZ for women exposed during the 3rd trimester of pregnancy, and over-estimated it for those exposed early in pregnancy. Nevertheless, to reduce risks of compromised foetal development, our results support minimising exposure to cotton harvesting throughout pregnancy.

In Pakistan, the agriculture sector is the main source of income for rural women, and 76% of rural women in Sindh participate in cotton harvesting [26]. The average reduction in LAZ of infants born to mothers who had worked in the cotton industry during pregnancy, in our study, was 0.35 SD, when both its direct and indirect effects are considered. This effect size rivals those of established interventions to prevent early childhood stunting, such as the promotion of exclusive breastfeeding, improved hygiene and sanitation practices or, in late infancy, feeding nutrient dense complementary foods [27]. It underscores a need to improve working conditions for pregnant women involved in cotton harvesting to reduce stunting in early infancy. It suggests the prevalence of stunting could increase in Sindh if the percentage of rural pregnant women harvesting cotton increases over time without improved working conditions.

The major strengths of this study include the collection of data over a relatively short period of time from a large representative sample of women who had recently given birth, which minimises errors in estimates of infant age and controls for seasonal factors influencing birth outcomes and early infant growth. Selection bias was also low (i.e. less than 1% of refusals; 1.3% and 1.6% of missing data for maternal BMI and infant LAZ, respectively, and 1.8% of missing data on infant LAZ after removal of outliers). Only three teams of well-trained and carefully selected anthropometrists made anthropometric measurements. Electronic data capture and regular monitoring at the point of data capture also ensured rigorous data quality control. To our knowledge, it is also the first study to examine the relationship between labour-intensive agriculture work and infant and maternal outcomes using structural-equation modelling to interpret interdependencies.

The results can be generalised to most villages in rural irrigated areas of Sindh, but they may not extend to other areas of Pakistan, especially areas where the prevalence of maternal underweight is lower than in Sindh. Our analyses were based on post-natal maternal BMI and infant LAZ at 2–12 weeks instead of pre-conceptual maternal BMI, weight gain in pregnancy and infant birth weights. The measurement of the exposure was based on a non-validated questionnaire for this population. Furthermore, the data were collected retrospectively, and the intensity of agricultural work (i.e. number of hours worked over the day) was not assessed. These underlying assumptions for maternal and infant anthropometric status, and potential errors in exposure measurement (recall bias and unaccounted variability) may attenuate relationships between agriculture work during pregnancy and maternal and infant anthropometric status. Recall bias, for participation (or not) in the three types of agriculture related activities, is likely to be low because roles are well-established, they occur over a relatively long period of time and cotton harvesting generates income. Finally, the cross-sectional nature of the study design does not establish causality.

Although the focus of our study was on exploring the relationship between agricultural work and nutrition outcomes, it is important that future studies investigate the relationship between different forms of work (i.e domestic and agricultural) and nutrition. This would require reliable time-use data.

## Conclusions

This study shows a very high prevalence of stunted growth among 2–12-week-old infants in irrigated areas of Sindh province Pakistan. Failure to address these high rates of stunting in early life will have both short and long-term negative consequences on health, cognitive development and adult stature, which in turn increases the risks for poor pregnancy outcomes and adult work capacity later in life [28]. Cotton harvesting during pregnancy was negatively associated with post-partum maternal BMI and infant LAZ, even after controlling for household wealth and education. Sixteen percent of the association between cotton harvesting and infant LAZ was mediated via its influence on maternal BMI.

Pakistan’s economy relies on the agriculture sector. Given that both the commercialisation of agriculture and women’s involvement in agriculture are increasing, the need to invest in rural women and their working conditions is urgent to improve the long-term health and nutritional status of rural populations. Future studies should evaluate the cost-benefits of alternative interventions designed to protect pregnant women working in commercial crop-related agriculture, especially cotton harvesting. Potential interventions include the provision of an energy and micronutrient dense food supplement to address maternal undernutrition, a cash incentive to allow pregnant women the choice of either withdrawing from physically-demanding agriculture work or reducing the number of hours worked; and perhaps the use of protective equipment, when cotton harvesting, to reduce pesticide exposure. Failure to improve these conditions for women during pregnancy, might limit efforts to achieve the global sustainable development goal of reducing childhood stunting in Pakistan.

## Supplementary information

**Additional file 1.** Sampling.

**Additional file 2.** Baseline survey questionnaire.

**Additional file 3.** Hypothesized models of pathways related to maternal BMI represented on a directed acyclic graph (DAG).

**Additional file 4.** Sample flow chart.

## Data Availability

The datasets used and analysed during the current study are available from the corresponding author on reasonable request.

## Abbreviations

BMI: Body mass index
CI: Confidence interval
DAG: Directed acyclic graphs
IQR: Inter-quartile range
LAZ: Length-for-age Z-score
LMIC: Low-and middle-income countries
Ref: Reference
SES: Socio-economic status
TEM: Technical error of measurement
WHO: World Health Organization
WLZ: Weight-for-length Z-score
