# Supplementary material for: Maternal BMI mediates the impact of crop-related agricultural work during pregnancy on infant length in rural Pakistan: a mediation analysis of cross-sectional data
Source: BMC Pregnancy Childbirth. 2019 Dec 17;19:504. doi: 10.1186/s12884-019-2638-3 (PMC6918638; doi:10.1186/s12884-019-2638-3)
Supplement: Supplementary file 1 — Additional file 1. Sampling. [file 12884_2019_2638_MOESM1_ESM.docx]

**Additional file 1: Sampling**

A sample size of 1000 dyads was chosen to detect a difference in maternal BMI of 0.18 for every additional hour worked with 80% power at a 5% level of significance (1). This sample size also provides adequate power to explore factors associated with maternal and infant nutritional status.

Participants were selected via systematic random cluster sampling. In the first phase, all administrative villages with perennial canal irrigation were selected (2,911 of 5,775 administrative villages). Villages with perennial canal irrigation were chosen as the study site because women in these villages are frequently involved in commercial agriculture, including cotton harvesting. The reported populations of these villages ranged from <100 to >34,000 and we therefore excluded villages if their population, as reported in the 1998 census, was below the 10^th^ and above the 90^th^ percentiles of estimated village sizes (i.e., populations < 1000 or > 7800). All eligible villages were listed in alphabetical ascending order by district (n=2329 villages); and every 37^th^ village was selected from a randomly selected start point in the list to provide an estimated sample size (based on birth rates) of 1000 mother-infant dyads. All dyads living in the selected villages were invited to participate in the study if they met the following inclusion criteria: (i) infant ≥2 weeks and ≤ 12 weeks of age on the day of the first interview; (ii) healthy infant without congenital deformations that would impact on their ability to eat; (iii) the primary caregiver (i.e. the biological mother) intends to reside in the study area over the next 10 months and (iv) a singleton birth.

To recruit these dyads, a community profiling procedure was used, in which local key informants, including: health workers, midwives, doctors, paramedics, and local authorities identified recent births. In the first stage, key informants were asked to list exhaustively all kinship groups/castes and localities within the village and then to list all births within the past three months from within those castes/localities. Probes were used to minimise omissions. A fieldworker visited each listed mother to confirm eligibility and probe for other births within the locality.

**References**

1. National Institute of Population Studies (NIPS) [Pakistan] and ICF International. Pakistan Demographic and Health Survey 2012–13. Islamabad, Pakistan and Calverton, Maryland, USA: NIPS and ICF International 2013.
