# Supplementary material for: Maternal BMI mediates the impact of crop-related agricultural work during pregnancy on infant length in rural Pakistan: a mediation analysis of cross-sectional data
Source: BMC Pregnancy Childbirth. 2019 Dec 17;19:504. doi: 10.1186/s12884-019-2638-3 (PMC6918638; doi:10.1186/s12884-019-2638-3)
Supplement: Supplementary file 2 — Additional file 2. Baseline survey questionnaire. [file 12884_2019_2638_MOESM2_ESM.docx]

| 26 Planting/weeding |
| --- |
| 27 Plucking/cleaning |
| 28 Seedling preparation |
| Health-related activities |
| 29 Visiting lady health worker |
| 30 Visiting medical facility |
| 31 **Travelling** |
| 94 Other (Specify) ____________ |
| 95 Other (Specify) ____________ |
| 96 Other (Specify) ____________ |

Q4. Record each of the activities listed above such as sleeping, resting, eating and personal care for each of one-hour intervals in the 24 hour cycles in the reference day. Ask for each hour and record the activities carried out in that hour. Multiple activities can be recorded within any one-hour interval. **If the respondent recalls any new activity not previously recorded in Q3, then first record it in relevant row of the table 3 above and then note its time interval in table 4**. Finally, record in whose care the reference child was during each one-hour interval.

Dummy question (1=Proceed)

Table 4

|  | Activities | 5.00-6:00 AM | 6:00-7.00 AM | And so on till 4.00-5.00 AM |
| --- | --- | --- | --- | --- |
|  |  |  |  |  |
| 1 | Sleeping and resting | □ | □ | □ |
| 2 | Personal care (bathing, dressing etc) | □ | □ | □ |
| 3 | Eating | □ | □ | □ |
| 4 | Other personal care (Specify) __________________ | □ | □ | □ |
| 5 | [Auto-generated code and activity name from table above] | □ | □ | □ |
| 6 | [Auto-generated code and activity name from table above] | □ | □ | □ |
| 7 | [Auto-generated code and activity name from table above] | □ | □ | □ |
| 8 | [Auto-generated code and activity name from table above] | □ | □ | □ |
| 9-onwards | [Auto-generated code and activity name from table above] and so on | □ | □ | □ |
| 4a | In whose care was the reference child?  01=Only self  02=Self and someone else  03=Only someone else  04=No one  98=Don‘t know |  |  |  |

# 12. Agriculture and land

*Instructions:* *Probe for and list all the land under 1.1 first. Then complete all of the questions from 1a-1i for each piece of land before proceeding to the next piece of land.*

*Read aloud:* Now, we would like information on your household’s agriculture-related behaviors and use of land.

| No. | Questions and filters | Coding categories | | SKIP |
| --- | --- | --- | --- | --- |
| 1. | Does any member of your household own agricultural land? | 01 = Yes  02 = No | | >Q1.1  >Skip to Q2 |
| 1z | Who in the household owns agricultural land? *For each person, insert roster code below. Then go through Q1a-1i for each person that owns agricultural land (i.e. column by column).* Only upto two perons who own agricultural land from the household can be listed | | |  |
|  | Roster code: | 1 □□ | 2 □□ |  |
| 1a | What is the size of the land in acres? | □□  98 = Don’t know | □□  98 = Don’t know |  |
| 1b | How much say do you have in what the land is used for? | 01 = Complete say on your own  02 = Some say in consultation with someone else  03 = Someone else decides but will consult you  04 = No say at all  88 = Did not answer  98 = Don’t know | 01 = Complete say on your own  02 = Some say in consultation with someone else  03 = Someone else decides but will consult you  04 = No say at all  88 = Did not answer  98 = Don’t know |  |
| 1c | How much say do you have in selling the land? | 01 = Complete say on your own  02 = Some say in consultation with someone else  03 = Someone else decides but will consult you  04 = No say at all  88=Did not answer  98 = Don’t know | 01 = Complete say on your own  02 = Some say in consultation with someone else  03 = Someone else decides but will consult you  04 = No say at all  88=Did not answer  98 = Don’t know |  |
| 1d | How did you/they come to own the land? | 01 = Inherited from family  02 = Bought the land  03 = Government allotment  96 = Other (specify) _____________ | 01 = Inherited from family  02 = Bought the land  03 = Government allotment  96 = Other (specify) _____________ |  |
| 1e | How much of this land is currently cultivated? (Acres) | □□  98= Don’t know | □□  98 = Don’t know |  |
| 1f | What was the main water source for this cultivated land?  *Multiple answers are possible*. *Do not read possible answers* | 01=Canal  02=Tubewell/Peter pump  03 = Rain water  04 = River/Stream  05= Pond  06=Well  96= Other (Specify)______ | 01=Canal  02=Tubewell/Peter Pump  03 = Rain water  04 = River/Stream  05= Pond  06=Well  96= Other (Specify)______ |  |
| 1g | Is any of this land being cultivated by a household member (cultivated alone or in partnership with someone)? | 01 = Yes  02 = No | 01 = Yes  02 = No | Q2 |
| 1h | What are the labour arrangements?  *Multiple answers are possible*. *Do not read possible answers* | 01 = family labour  02 = casual hired labour  03 = Farm employees  04 = reciprocal exchange labour  96=Other(Specify) ___________ | 01 = family labour  02 = casual hired labour  03 = Farm employees  04 = reciprocal exchange labour  96=Other(Specify) __________ |  |
| 1i | Which crops are being grown on it?  *See codes below*  *Multiple answers are possible. Do not read possible answers* | □□ | □□ |  |

| No. | Questions and filters | Coding categories | | SKIP |
| --- | --- | --- | --- | --- |
| 2. | Is any member of your household cultivating any land which did not belong to the household? | 01 = Yes  02 = No | | >Q2.1  >Skip to next section |
|  | Who in the household cultivates agricultural land that does not belong to the household? *For each person, insert roster code below. Then go through 2a-2e for each person (i.e. column by column).* | | |  |
| 2z | Roster code: | 1 □□ | 2 □□ |  |
| 2a | How much land? (Acres) | □□  98 = Don’t know | □□  98 = Don’t know |  |
| 2b | What was the arrangement? | 01 = Sharecropping half share to tenant  02 = Sharecopping quarter share to tenant  03 = Sharecropping (other specify)____________  04 = Fixed rent  96 = Other (specify) ________ | 01 = Sharecropping half share to tenant  02 = Sharecopping quarter share to tenant  03 = Sharecropping (other (specify)____________  04 = Fixed rent  96 = Other (specify) ________ |  |
| 2c | What was the main water source for this crop?  *Multiple answers are possible. Do not read possible answers* | 01=Canal  02=Tubewell/Peter Pump  03 = Rain water  04 = River/Stream  05= Pond  06=Well  96= Other (Specify)______ | 01=Canal  02=Tubewell/Peter Pump  03 = Rain water  04 = River/Stream  05= Pond  06=Well  96= Other (Specify)______ |  |
| 2d | What are the labour arrangements?  *Multiple answers are possible. Do not read possible answers* | 01 = family labour  02 = casual hired labour  03 = farm employees  04 = reciprocal exchange labour  96=Other(Specify) ______ | 01 = family labour  02 = casual hired labour  03 = farm employees  04 = reciprocal exchange labour  96=Other(Specify) _______ |  |
| 2e | Which crops are being grown on it?  See codes below  *Multiple answers are possible. Do not read possible answers* | □□ | □□ |  |

| **Codes for 1i and 2e** | | |
| --- | --- | --- |
| 01= Wheat | 25=Chilli | 49=Lychee |
| 02 = Sugarcane | 26=Egg plant | 50=Apple |
| 03 = Rice | 27=Okra/lady finger | 51=Peach |
| 04 = Cotton | 28=Green leaves | 52=Plum |
| 05 = Tobacco | 29=Onion | 53=Berries |
| 06 = Maize | 30=Potatoe | 54=Pineapple |
| 07 = Gram | 31=Tomatoes | 55= Sapodilla (cheeku) |
| 08 = Jowar | 32=Capsicum | 96=Other |
| 09 = Barley | 33=Pumpkin/zuccini |  |
| 10 = Bajra | 34=Cauliflour |  |
| 11 = Rapeseed & Mustard | 35=Bottle gourd |  |
| 12 = Sunflower | 36=Sponge guord |  |
| 13 = Soybean | 37=Bitter gourd |  |
| 14 = Linseed | 38 = Green beans |  |
| 15 = Sesasum | 39=Garlic |  |
| 16 = Fodder | 40=Ginger |  |
| 17 = Mattar | 41=Avacado |  |
| 18 = Mash | 42=Banana |  |
| 19 = Mung | 43=Guava |  |
| 20 = Masoor | 44=Mango |  |
| 21 = Pulses (other than listed) | 45=Lemon/lime |  |
| 22=Cabbage | 46=Orange/tangerine |  |
| 23=Carrot | 47=Papaya |  |
| 24=Cucumber | 48=Melon |  |

#

# 13. Livestock ownership

*Instructions:*

1.Does your household and/or any of members own fully or on any shared basis any of the following types of animal/poultry?

|  | 01= Yes 02=No |
| --- | --- |
| 01=Goats/kid |  |
| 02=Sheep/lamb |  |
| 03=Cow /calf/bull |  |
| 04=Buffalo/calf |  |
| 05=Chickens/poultry/ducks |  |

If No in all categories, skip to next section. If Yes in any category ask Q 1a-1h for that category. [Category code and type will be autogenerated]

|  | Animal code | Auto-generated | [sequence] |
| --- | --- | --- | --- |
| 1z | Animal | Auto-generated | [sequence] |
| 1a | How many ______ does your household currently own outright? If none write “0” and go to Q 1c |  |  |
| 1b | Of these how many are owned by you? |  |  |
| 1c | How many ______are kept on a shared basis? |  |  |
| 1d | Of these how many are shared by you? |  |  |
| 1e | What is the purpose for keeping the animals?  *Multiple answers are possible. Do not read possible answers*  01 = Milk for household  02=Milk for selling  03= Cash from sales (now or in future)  04= Dowry  05 = Meat for household  06=Meat for selling  07 = Egg for household  08=Eggs for selling  09 = Manure  96 = Other specify ___________ |  |  |
| 1f | Does a household member or a non-household member usually takes care of the animals?  01-Household member  02=Non-household member>>1h |  |  |
| 1g | Which household member?  Insert roster code  *Multiple answers are possible. Do not read possible answers* |  |  |
| 1h | How much say do you have in the sale/purchase of livestock that you own?  01 = Complete say on your own  02 = Some say in consultation with someone else  03 = Someone else decides but will consult you  04 = No say at all  98 = Don’t know |  |  |
| 1i | How much say do you have in the use of cash earned from the sale of livestock or livestock products?  01 = Complete say on your own  02 = Some say in consultation with someone else  03 = Someone else decides but will consult you  04 = No say at all  98 = Don’t know |  |  |

#

# 14. Social assistance

1. Did you or your household receive any type of cash/assistance from any source during the past year?

01=Yes >> Q1.1

02=No >>Next section

| No. |  | 1z | 1a | 1b | 1c | 1d | 1e |
| --- | --- | --- | --- | --- | --- | --- | --- |
|  | Item Categories | In the last 12 months have you or your household received_____?  01 = Yes > Go to Q1a  02 = No >*Go to next item* | Did you (survey mother) receive this assistance?  01=Yes  02=No | Is the assistance being currently received?  01=Yes  02=No | Amount in rupees received last time? | Spent on what? (Multiple uses are possible. *Do not read possible uses*)  *Code list below*  01 = Buy food for household consumption  02 = Buy food specifically for children  03 = Buy non-food items for yourself  04 = Buy non-food items for others or for the household  05 = Buy non-food items specifically for children  06 = Spent on health or illness  07=Spent on transportation  08=Spent on school fees and books  09=Saved  96=Other (Specify)______ | Who made the decisions on how to spend it?  01 = Complete say on your own  02 = Some say in consultation with someone else  03 = Someone else decided but consulted you  04 = No say at all  98 = Don’t know |
| 1 | Cash transfer from BISP | □□ |  | □□ | □□ |  |  |
| 2 | Official Zakat | □□ |  | □□ | □□ |  |  |
| 3 | Private zakat or charity | □□ |  | □□ | □□ |  |  |
| 4 | Remittance from household member | □□ |  | □□ | □□ |  |  |
| 5 | Microcredit | □□ |  | □□ | □□ |  |  |
| 6 | Support from NGOs | □□ |  | □□ | □□ |  |  |
| 95 | Other government support  (Specify)_______________ | □□ |  | □□ | □□ |  |  |
| 96 | Other support  (Specify)_______________ | □□ |  | □□ | □□ |  |  |

#

# 15. Household facilities and ownership

Subsection A: Facilities

*Read aloud:*

Now we have some questions about your housing and assets. Please be reassured that we will not share this information with anyone else and it will be treated as confidential.

| No. | Questions and filters | Coding categories | SKIP |
| --- | --- | --- | --- |
| 1 | Is the house you live in, your own, rented or do you stay with someone else? | 01 = Own  02 = Rent 03 = Live with someone else (no rent)  04 = Government  88 = Refused 96 = Other (specify)____________________  98 = Don’t know |  |
| 2 | How many rooms in your house are used for sleeping? | No. of rooms □□  88 = Refused  98 = Don’t know |  |
| 3 | Does this house have an electricity connection? | 01 = Yes  02 = No  88 = Refused  98 = Don’t know |  |
| 4 | What is your main source of energy for cooking? | 01 = Electricity  02 = Liquefied propane gas  03 = Natural gas  04 = Biogas  05 = Firewood  06 = Kerosene  07 = Charcoal/coal/lignite  08 = Animal Dung  09 = Dried leaves/shrub  10 = Agricultural crop/straw  96 = Other (Specify) _________________ |  |
| 5. | What is the main material used to construct the floor of the house? | 01 = Cement  03= Tile  04 = Marble  05 = Mud  06=Bricks  96 = Other (specify)____________________  98 = Don’t know |  |
| 5a | Is the main material used to construct floor of the house observed and verified by the enumerator? | 01=Yes  02=No |  |
| 6. | What is the main material used to construct the ceiling/roof of the house? | 01 – Reinforced Concrete(RCC )/Cement  03 = Iron sheets  04 = Bamboo/twigs  05 = T-Iron/Wood  06 = Tile beam  07 = Cardboard/plastic  08 = No ceiling  96 = Other (specify)_____________________  98 = Don’t know |  |
| 6a | Is the main material used to construct the ceiling/roof of the house observed and verified by the enumerator? | 01=Yes  02=No |  |
| 7. | What (main) material has been used to construct most of the walls of the house? | 01 = Cement blocks  03= Burned bricks  04 = Unbaked bricks  05 = Wood  06 = Stone  07 = Mud  08=Bamboo  96 = Other (specify)_____________________  98 = Don’t know |  |
| 7a. | Is the main material used to construct most of the walls of the house observed and verified by the enumerator? | 01=Yes  02=No |  |

Subsection B: Assets

*Read Aloud:* Now I would like to ask you some information regarding specific assets you may own in your household.

Q1. *Read each asset one by one. If the household does not have the particular asset, please record ‘0’. Please do not count any devices more than once, even if this device has multiple functions (e.g. television that is also used as a radio).*

Dummy question (1=Proceed)

| 1z Assets |  |
| --- | --- |
|  | 1a. How many _____________ do you or any member of your household own? |
| i. Sewing machine | □□ |
| ii. Electric Fan- (Ceiling, Table, Pedestal, Exhaust) | □□ |
| iii. Air Conditioner | □□ |
| iv.Refrigerator | □□ |
| v. Television | □□ |
| vi. Dish/antenna | □□ |
| vii. CD/DVD player | □□ |
| viii. Car/Datsun/Truck | □□ |
| ix. Bicycle | □□ |
| x. Motorcycle/Scooter | □□ |
| xi. Mobile Phone | □□ |
| xii. Computer | □□ |
| xiii. UPS/Generator | □□ |
| xiv. Animal-drawn cart | □□ |
| xv. Tractor | □□ |
| xvi. Qingqi/rickshaw | □□ |
| xvii. Donkey | □□ |
| xviii. Mule | □□ |
| xix. Horse | □□ |
| xx. Camel | □□ |
| xxi. Bull | □□ |

#

# 16. Water, sanitation and hygiene

Subsection A: Water

| No. | Questions and filters | Coding categories | SKIP |
| --- | --- | --- | --- |
| 1. | What is the main source of drinking water for your household? | 01 = Piped water  02 = Hand pump  03 = Tube well/Motorized pumping  04 = Open Well  05 = Closed well  06=Pond/canal/river/stream  07 = Spring  08= Mineral water  09=Tanker truck/Water bearer  10=Filtration plant  88 = Refused 96 = Other (specify)____________________  98 = Don’t know |  |
| 2. | Do you treat your water in any way to make it safer to drink? | 01 = Yes  02 = No  88=Refused  98 = Don’t know | Q3  Q4  Q4  Q4 |
| 3. | What do you usually do to treat the water to make it safer to drink?  *Multiple answers possible. Don’t read possible answers.*  Anything else? Record all items mentioned | 01 = Boil  02 = Add bleach/chlorine  03=Add alum  04 = Strain it through a cloth  05 = Use a water filter  06 = Solar disinfection  07 = Let it stand and settle  96 = Other (specify) ____________________  98 = Don’t know |  |
| 4. | Is the drinking water pot covered? | 01 = Yes  03= No  04 = N/A – Water not kept in water pot |  |
| 4a. | Observed and verified by the enumerator? | 01=Yes  02=No |  |

Subsection B: Sanitation

| No. | Questions and filters | Coding categories | | SKIP |
| --- | --- | --- | --- | --- |
| 1. | What kind of toilet facility do members of your household usually use?  If ‘flush’ or ‘pour flush’ probe: Where does it flush too? | | *Flush or Pour Flush Toilet*  01 = Flush to piped sewer system  02 = Flush to septic tank  03 = Flush to pit latrine  04 = Flush to somewhere else  05= Flush, don't know where  *Pit Latrine*  06 = Ventilated improved pit latrine  07 = Pit latrine with slab  08 = Pit latrine without slab/Open pit  09 = No facility/bush/field  96 = Other (Specify) _________________. |  |
| 2. | What material has been used to construct the floor of the toilet/latrine area? | | 01 = Cement  02 = Tile  03 = Marble  04 = Mud  88 = Refused  96 = Other (specify)______________________  98 = Don’t know |  |
| 3 | **Observation only**  Is there any soap next to toilet/latrine? | | 01 = Yes  02 = No  03=Not observed |  |
| 4 | What material has been used to construct the floor of the kitchen/cooking area? | | 01 = Cement  02 = Tile  03 = Marble  04 = Mud  88 = Refused  96 = Other (specify)_____________________  98 = Don’t know |  |
| 5 | **Observation only**  Is the kitchen/cooking area clean? | | 01 = Yes  02 = No  03= Could not be observed |  |
| 6 | **Observation only**  Is there any soap next to the kitchen/cooking area? | | 01 = Soap present  02 = Soap absent  03= Could not be observed |  |
| 7 | **Observation only**  Is the dishware clean? | | 01 = Yes  02 = No  03= Could not be observed |  |
| 8 | **Observation only**  See the location of kitchen/cooking area and bathroom/latrine | | 01 = Separate and not in sight of each other  02 = Separate but in sight of each other  03 = Not separate  04= Could not be observed |  |
| 9 | What did you use soap for yesterday?  Multiple answers possible. Don't read possible answers. | | 01 = Washing clothes  02 = Washing own body  03 = Washing child’s body/bottom  04 = Washing hands after defecating  05 = Washing hands after cleaning child  06 = Washing hands before feeding a child  07 = Washing hands before cooking  08 = Washing hands before eating  09 = Washing hands after eating  10 = Washing child's hands before eating  11 = Washing child's hands after eating  12 = Didn't use soap yesterday  88 = Refused  96 = Other (specify)____________________  98 = Don’t know |  |

Subsection C: Hygiene

| No. | Questions and filters | Coding categories | SKIP |
| --- | --- | --- | --- |
| 1. | Where do you dispose of household waste?  Multiple answers possible. Don’t read possible answers. | 01= Garbage pit  02 = In the kitchen garden  03 = Thrown out indiscriminately  04 = On the street  05 = Open space  06 = Composting  96 = Other (specify)___________________  98 = Don’t know |  |
| 2. | Is there animal waste (livestock, poultry, pets etc.) around the house or in the compound? | 01=Yes, around the house and in the compound  03 = No  04 = Yes, around the house only  05 = Yes, in the compound only |  |
| 2a | Observed and verified by the enumerator? | 01=Yes  02=No |  |
| 3. | Are there animals inside the house/in the same compound where people cook and eat? | 01 = Yes  03 = No |  |
| 3a. | Observed and verified by the enumerator? | 01=Yes  02=No |  |
| 4. | Is there any instrument of protection of food against flies? | 01 = Yes  03 = No |  |
| 4a | Observed and verified by the enumerator? | 01=Yes  02=No |  |
| 5. | Is there an open sewerage drain, and/or any place for open defecation close to your home?  Within 100 metres or within visible sight of home | 01 = Yes  03 = No  98 = Don’t know |  |
| 5a | Observed and verified by the enumerator? | 01=Yes  02=No |  |
