# Supplementary material for: Maternal BMI mediates the impact of crop-related agricultural work during pregnancy on infant length in rural Pakistan: a mediation analysis of cross-sectional data
Source: BMC Pregnancy Childbirth. 2019 Dec 17;19:504. doi: 10.1186/s12884-019-2638-3 (PMC6918638; doi:10.1186/s12884-019-2638-3)
Supplement: Supplementary file 3 — Additional file 3. Hypothesized models of pathways related to maternal BMI represented on a directed acyclic graph (DAG). [file 12884_2019_2638_MOESM3_ESM.docx]

**Additional file 3:** **Hypothesized models of pathways related to maternal BMI represented on a directed acyclic graph (DAG)**

*Model 1: testing the relationship between*


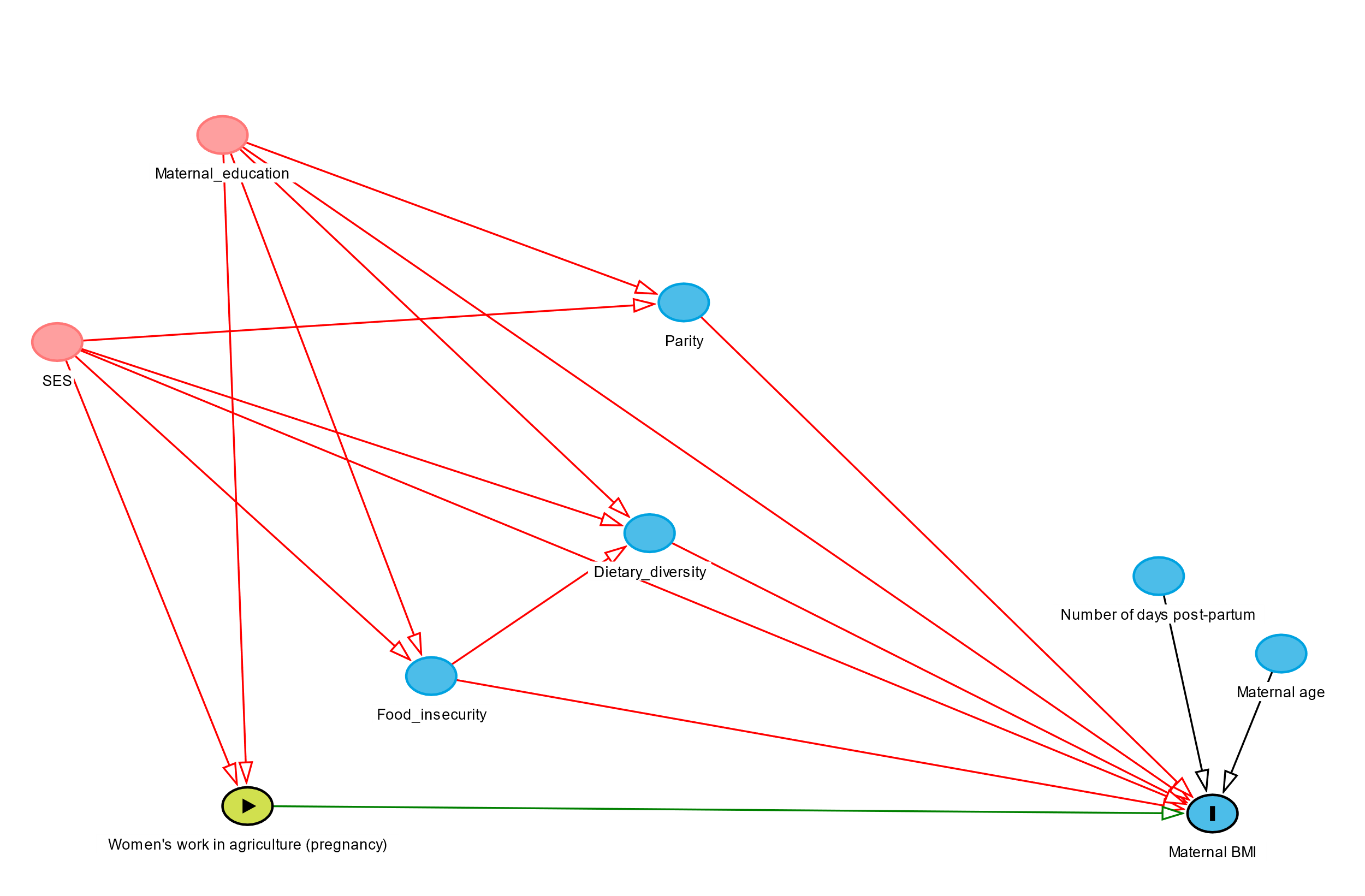


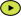
exposure
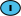
outcome
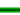
causal path
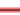
biasing path
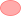
ancestor of exposure and outcome
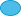
ancestor of outcome
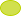
ancestor of exposure

**Appendix 3: Hypothesized models of pathways related to infant size represented on a directed acyclic graph (DAG) (continued)**

*Model 2: testing the relationship between and infant LAZ*


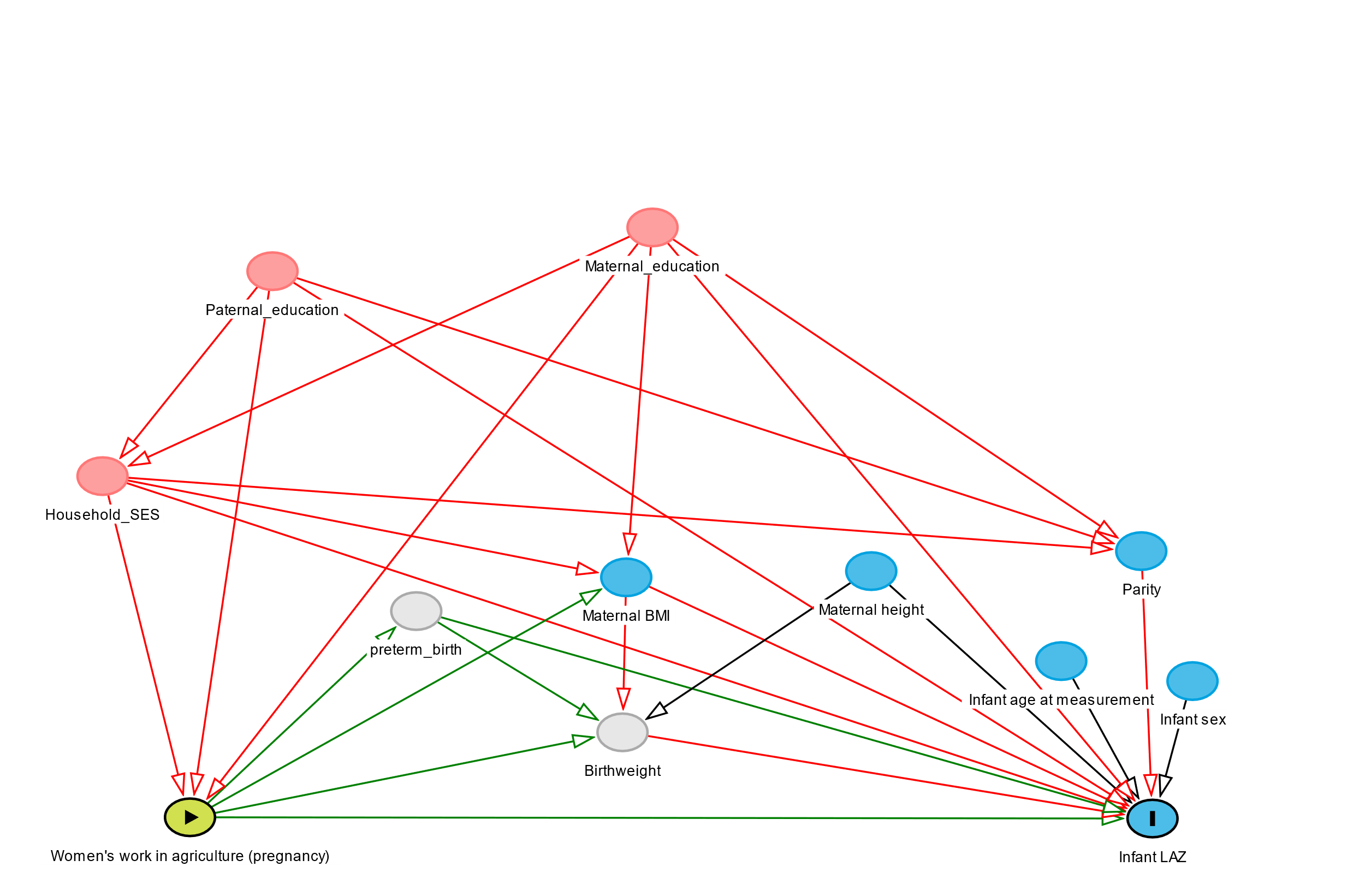


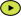
exposure
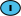
outcome
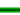
causal path
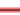
biasing path
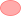
ancestor of exposure and outcome
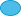
ancestor of outcome
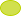
ancestor of exposure
