# Supplementary material for: Maternal BMI mediates the impact of crop-related agricultural work during pregnancy on infant length in rural Pakistan: a mediation analysis of cross-sectional data
Source: BMC Pregnancy Childbirth. 2019 Dec 17;19:504. doi: 10.1186/s12884-019-2638-3 (PMC6918638; doi:10.1186/s12884-019-2638-3)
Supplement: Supplementary file 4 — Additional file 4. Sample flow chart. [file 12884_2019_2638_MOESM4_ESM.docx]

**Additional file 4: Sample flow chart**

Ineligible:

- Urban areas
- Non-irrigated areas
- Villages with a population below the 10^th^ and above the 90^th^ percentiles of estimated village sizes

**(n=3,446)**

Women’s Work in agriculture and Nutrition study –Villages in Sindh province **(n=5,775)**

Excluded:

- Missing

- Maternal BMI (n=15)

- Infant LAZ (n=18)

- Outliers

- Infant LAZ (n=9)

**(n=15 for mothers; n=27 for infants)**

mother-infant dyads **(n=2,329)**

Final analysis sample (mother-infant dyads) **(n=1146 for mothers; n=1134 for infants)**

Final study sample (mother-infant dyads) **(n=1,161)**

Ineligible/refusals:

- Babies <2 weeks and >12 weeks
- Babies with congenital deformation
- Primary caregiver not the biological mother
- Primary caregiver does not intend to reside in the area for more than 10 months
- Refusals

**(n=568)**

Final sample of rural villages included for this study **(n=62).** Within these villages, all births within 2-12 weeks were recruited. Eligible mothers **(n=1729)**

Excluded:

Rural villages not randomly selected to recruit 1,000 mother-infant dyads

**(n=2,267)**

mother-infant dyads **(n=2,329)**

Eligible rural villages for this study **(n=2,329)**
